# Prognostic and clinicopathological significance of CD133 in patients with hepatocellular carcinoma: A meta-analysis

**DOI:** 10.1097/MD.0000000000048389

**Published:** 2026-04-24

**Authors:** Baoguo Kang, Dandan Liang, Dajie Liu

**Affiliations:** a Department of Oncology, Liangjiang Hospital of Chongqing Medical University (People’s Hospital of Chongqing Liangjiang New Area), Chongqing, China; b Division of Infectious Diseases, Chongqing Public Health Medical Center, Chongqing, China; c Department of Oncology, The Sixth People’s Hospital of Chongqing, Chongqing, China.

**Keywords:** Cancer stem cell, CD133, hepatocellular carcinoma, meta-analysis, prognosis, systematic review

## Abstract

**Background:**

This meta-analysis was conducted to investigate whether CD133 could be used as a biomarker to predict prognosis in hepatocellular carcinoma patients.

**Methods:**

We systematically retrieved relevant studies from these databases as of February 2026. Odds ratios and their 95% confidence intervals were used to estimate the association between CD133 expression and clinicopathological features. The associations between CD133 and survival outcomes, including overall survival (OS), disease-free survival, and recurrence-free survival, were estimated by hazard ratios and 95% confidence intervals.

**Results:**

A total of 25 publications were included, involving 3278 patients. CD133 was highly expressed in liver cancer tissues (*P* < .05). Also, CD133 expression was associated with tumor size, tumor node metastasis stage, and tumor differentiation (*P* < .05). CD133-positive expression had shorter OS, disease-free survival, and recurrence-free survival than those with CD133-negative expression (*P* < .05).

**Conclusion:**

This meta-analysis suggests that CD133 is a potential biomarker for predicting poor survival in Asian hepatocellular carcinoma patients, especially for OS.

## 1. Introduction

Hepatocellular carcinoma (HCC) is a malignant tumor occurring in the liver with a high recurrence rate and high chemical resistance.^[1–3]^ According to the latest global analysis, liver cancer is one of the 3 major causes of cancer death in 46 countries, and new cases and related mortality are expected to rise sharply.^[4]^ It is estimated that 1.4 million cases will be diagnosed by 2040, and the number of new cases of liver cancer will increase by 55% compared with 2020. In addition, 1.3 million people will die of liver cancer, an increase of 56.4 percent over 2020. Although there are many treatments available, surgery and liver transplantation remain the most effective options.^[5]^ However, due to frequent postoperative recurrence and metastasis, the overall survival (OS) prognosis of HCC is poor.^[6]^ According to statistics, the recurrence rate of HCC is as high as 60–70%.^[7,8]^ The recurrence rate was as high as 43.1% within 2 years after hepatectomy, and the 5-year survival rate was only 30–50%.^[9,10]^ Therefore, it is of great significance to find reliable biomarkers to improve the prognosis and quality of life of HCC patients.

Cancer stem cells (CSCs) are a subset of tumor cells that exhibit dynamic characteristics. Its characteristic lies in its ability to self-renew, its capacity to differentiate into various different cell types, and its ability to trigger the formation of tumors.^[11,12]^ CSCs have been identified in a variety of solid tumors, including breast cancer, esophageal cancer, bladder cancer, lung cancer and colon cancer.^[13–17]^ More and more evidence indicates that CSCs play a significant role in cancer metastasis, poor prognosis, and resistance to conventional chemotherapy and radiotherapy.^[18–21]^ The tumor microenvironment maintains the characteristics of CSCs through factors such as hypoxia, interactions between immune cells, and secretion of factors, thereby creating a protective microenvironment that shields CSCs from the effects of treatment interventions. Under this framework, CD133 has become one of the most widely studied surface markers for CSCs, and is closely related to tumor origin, self-renewal and treatment resistance in various cancer types.^[22]^ Therefore, understanding the functional dynamics of CSCs and the role of markers such as CD133 is of vital importance for developing effective prognostic strategies and targeted treatment plans.

CD133 is a marker of CSC and has been used to identify CSC in a variety of tumors.^[23]^ Recent studies have shown that CD133 is an independent prognostic factor for several cancers, such as gastric cancer, non-small cell lung cancer, and colorectal cancer.^[24–26]^ Furthermore, high CD133 expression was associated with poorer survival.^[27]^ However, the use of CD133 to assess clinical outcomes in HCC patients remains controversial. Although published literature shows that CD133 is highly expressed in HCC, which is related to the poor prognosis of HCC patients.^[28–30]^ However, several studies have found that the high expression of CD133 is not related to the prognosis of HCC patients.^[31–36]^ Previous meta-analyses have explored the prognostic role of CD133 in HCC.^[37,38]^ However, there are limitations such as a limited sample size and the failure to analyze recurrence-free survival (RFS), etc. Therefore, in order to explore the clinical prognostic value of CD133 in HCC, we conducted a update meta-analysis of related clinical studies, aiming to provide more reliable evidence for basic research and clinical work.

## 2. Materials and methods

This study was performed in accordance with the Preferred Reporting Items for Systematic Reviews and Meta-Analyses guideline.^[39,40]^

### 2.1. Literature search

The 2 investigators used PubMed, EMBASE, Web of Science, CNKI, Google Scholar, and Cochrane Library databases to search the published literature on CD133 for prognoses in HCC patients. The period for retrieving literature was from the creation of the database until we systematically retrieved relevant studies from these databases as of February 2026. The following keywords and search terms were applied: “liver neoplasms,” “hepatocellular carcinoma,” “HCC,” “CD133,” “prognosis,” “survival,” etc (Table S1, Supplemental Digital Content, https://links.lww.com/MD/R762.).

### 2.2. Inclusion and exclusion criteria

Included studies should meet the following criteria:

1. Patients with HCC were diagnosed using pathology or histology.
2. Patients were divided into 2 groups based on CD133 expression.
3. Hazard ratios (HRs) and relevant 95% confidence intervals (CIs) are provided.
4. Survival outcomes, including OS, disease-free survival (DFS), and RFS, were reported.
5. Studies comparing the relationship between CD133 and clinicopathological features or prognosis were conducted.

The exclusion criteria are as follows:

1. Duplicate published studies.
2. Reviews, meta-analyses, conference abstracts, and case reports.
3. The subjects were not humans.
4. There is a dearth of studies with relevant data.

### 2.3. Data extraction

The 2 investigators extracted data from the included literature according to the self-designed table and cross-checked the data. The extracted information is as follows: first author, date of publication, country, sample size, age, detection method for CD133, threshold, treatment method for HCC, follow-up time, clinicopathological features, survival indicators (OS, DFS, RFS), HR and 95% CI, etc. HR and 95% CI were obtained directly from the literature. If HR and 95% CI were not available in the literature, Engauge Digitizer software was used to extract survival data from Kaplan–Meier curves, based on the approach described by Tierney et al.^[41]^ For the disputed data, the 2 investigators reached an agreement through discussion.

### 2.4. Quality assessment

The Newcastle-Ottawa Scale (NOS) is used to assess the quality of included studies.^[42]^ The total NOS ranges from 0 to 9 points. A NOS score over or equal to 7 points indicates high-quality studies.

### 2.5. Statistical analysis

The researcher directly obtained and calculated the HR for the effect size and its 95% CI from the included literature. HR > 1 suggests that CD133 overexpression is associated with a poor prognosis for HCC. The odds ratio (OR) and its 95% CIs estimated the association between CD133 expression and clinicopathological features. The chi-square test and *I*^*2*^ were used to examine the heterogeneity of the included studies. If *I*^*2*^ > 50% or *P* < .1, it is considered that there is heterogeneity between studies, and a random effect model is used to combine the effects.^[43]^ Otherwise, a fixed effect model is used. Subgroup analysis was performed based on sample size, treatment method, and follow-up time. A sensitivity analysis was carried out to assess the robustness of survival outcomes obtained through a one by one elimination method. If no fewer than 10 articles were included, Egger test and Begg test were used to explore potential publication bias.^[44]^ The significance level was set at *P < .05*. Statistical analysis was performed using Stata 14.0 software.

## 3. Results

### 3.1. Description of studies and quality assessment

**Figure 1.**
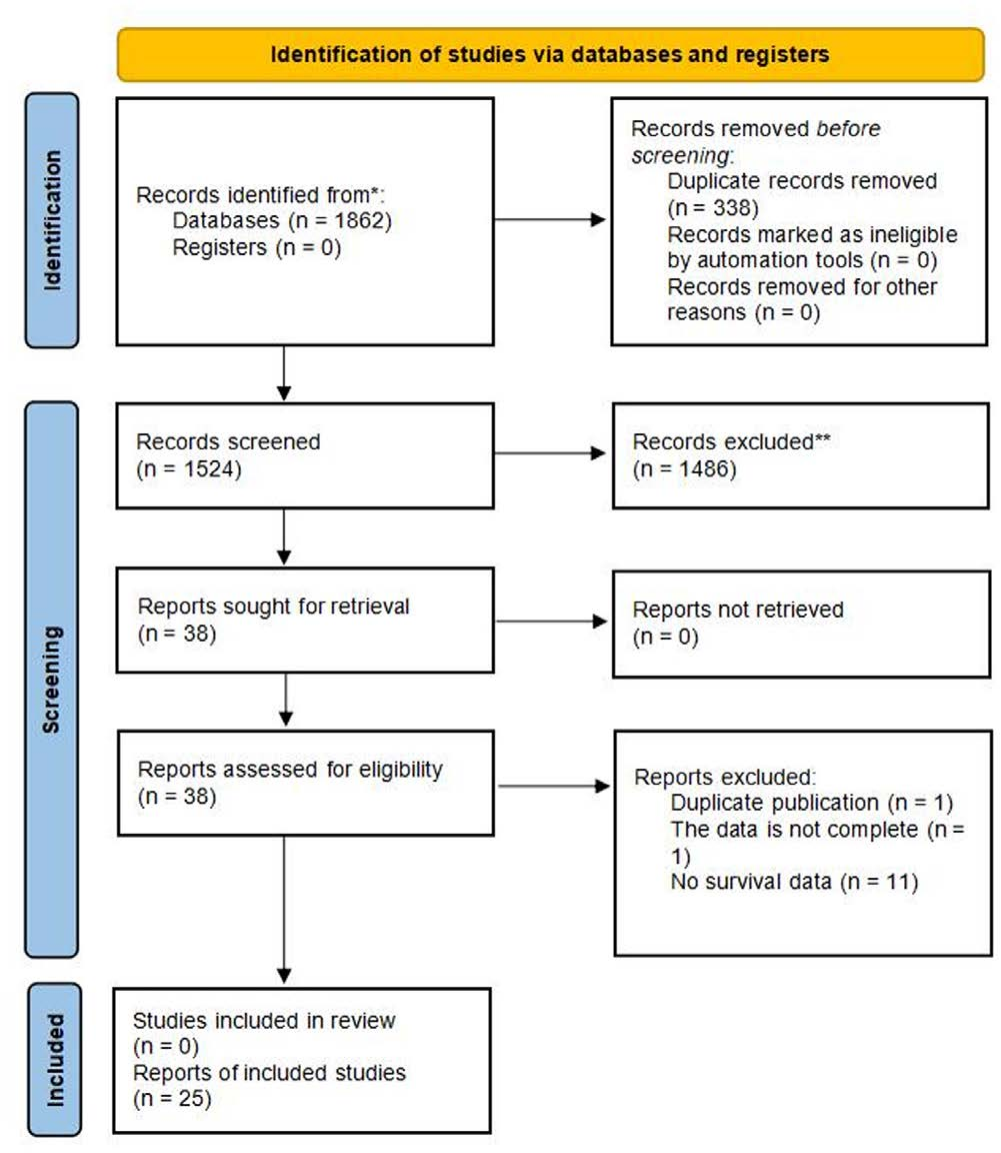
A PRISMA flow diagram of the literature search. n = number of records, PRISMA = Preferred Reporting Items for Systematic Reviews and Meta-Analyses.

By searching various databases, 1862 articles were obtained. After the deletion of duplicates and careful screening, a total of 3278 HCC patients were included in the meta-analysis in 25 studies.^[28,29,31–36,45–61]^ The screening process is shown in Figure 1. Twenty-three studies were from China. The number of patients in each study ranged from 35 to 387. Twenty-three studies reported the use of surgery; 1 study reported the use of surgical combination transhepatic arterial chemotherapy and embolization (TACE); and 1 study reported the use of transcatheter arterial embolization or TACE. OS was reported in 20 studies, DFS in 5 studies, and RFS in 3 studies. The total NOS score is 5 to 7. The basic features of the included studies are shown in Table 1.

**Table 1:** Characteristics of the included studies.

Studies	Origin of population	Sample size	Age(mean/median)	Technology	Cutoff values	Treatment	Follow-up (months)	NOS scores	Outcomes
Pan et al 2012	China	70	55	IHC	≥10 %	Surgery	62	5	OS
Wu et al 2013	China	190	59	IHC	Strong staining	Surgery	64 (Median follow-up time)	6	OS
Xie 2015	China	70	59	IHC	≥Score 1	Surgery	NR	7	OS
Liu 2013	China	245	48	IHC	≥Score 3	Surgery	80	6	OS
Jing et al 2019	China	43	62	IHC	≥30 %	Surgery	36	7	OS
Chen et al 2014	China	387	47	IHC	Strong staining	Surgery + TACE	96	6	OS
Song et al 2008	China	63	50.3	IHC	≥1.32 %	Surgery	60	6	OS, DFS
Yeh et al 2009	China	154	56.2	IHC	NR	Surgery	125	6	OS, DFS
Sasaki et al 2010	Japan	136	61	IHC	> 0%	Surgery	90	6	OS, DFS
Chan et al 2014	China	282	55.4	IHC	≥Score 1	Surgery	240	5	OS, DFS
Guo et al 2014	China	90	NR	IHC	≥5 %	Surgery	NR	6	OS
Zhao et al 2016	China	74	45.2	IHC	> 0%	Surgery	84	7	OS
Chen et al 2017	China	119	NR	IHC	Mean score	Surgery	33 (Median follow-up time)	6	OS, RFS
Dai et al 2018	China	127	57.3	IHC	≥Score 2	Surgery	215	7	OS, RFS
Xu et al 2018	China	108	54.87	IHC	≥Score 3	Surgery	NR	7	OS
Yu et al 2018	China	93	54	IHC	≥10 %	Surgery	NR	6	OS
Gong et al 2022	China	200	NR	IHC	≥Score 2	Surgery	NR	7	OS
Tseeleesuren et al 2022	China	120	58	RT-PCR	≥1.5	TAE/TACE	47.01 (Median follow-up time)	7	OS
Song et al 2017	China	89	57	IHC	> 0%	Surgery	NR	6	NR
Cui 2010	China	54	54	IHC	≥Score 3	Surgery	NR	7	NR
Zhao 2014	China	38	47	IHC	≥Score 3	Surgery	NR	6	NR
Yilmaz et al 2014	Turkey	35	64.3	IHC	≥Score 1	Surgery	NR	6	NR
Guo et al 2011	China	84	46.9	IHC	> 10 %	Surgery	NR	6	NR
Wan 2016	China	93	NR	IHC	≥10 %	Surgery	NR	7	OS, DFS
Yang et al 2010	China	314	NR	IHC	NR	Surgery	130	5	OS, RFS

DFS = disease-free survival, IHC = immunohistochemistr, NOS = Newcastle-Ottawa Quality Assessment Scale, NR = not reported, OS = overall survival, RFS = recurrence-free survival, RT-PCR = reverse transcription-polymerase chain reaction, TACE = transhepatic arterial chem otherapy and embolization, TAE = Transcatheter arterial embolization.

### 3.2. Association of CD133 expression with clinicopathological features

CD133 was highly expressed in HCC tissues with statistically significant differences (OR = 4.17, 95% CI (1.90, 9.14), *P < .0001*) (Table 2). Meanwhile, we found that CD133 expression was associated with tumor size, tumor node metastasis (TNM) stage, and tumor differentiation, with statistically significant differences (tumor size [OR = 1.29, 95% CI = 1.02, 1.64], *P* = .037; TNM stage [OR = 0.31, 95% CI = 0.14, 0.68], *P* = .003; and tumor differentiation [OR = 0.24, 95% CI = 0.08, 0.76], *P* = .015) (Table 2).

**Table 2:** Relationship between CD133 overexpression and the clinicopathologic features.

Variable	No. of studies	No. of patients	Heterogeneity		Effects model	OR (95%CI)	*P*
			*I*^*2*^ (%)	*P*			
Tumor tissue vs Para-carcinoma tissue	6	932	77.80	< .0001	Random	4.17 (1.90, 9.14)	< .0001
Age (50 > vs ≤ 50 years)	7	561	0	.624	Fixed	0.97 (0.68, 1.40)	.887
Gender (Male vs Female)	16	1947	10.90	.329	Fixed	1.20 (0.93, 1.54)	.165
HBV (Yes vs No)	7	1067	73.40	.001	Random	1.89 (0.94, 3.81)	.074
AFP (> 400 vs ≤ 400 ng/ml)	7	838	27.70	.217	Fixed	1.21 (0.91, 1.60)	.188
Tumor size (> 5 vs ≤ 5 cm)	14	1298	37.60	.083	Fixed	1.29 (1.02, 1.64)	.037
Tumor number (Single vs Multiple)	7	774	50.60	.059	Random	0.74 (0.45, 1.23)	.247
Lymphatic metastasis (Yes vs No)	4	617	69.80	.019	Random	1.25 (0.32, 4.81)	.750
Tumor capsule (Yes vs No)	6	499	68.90	.007	Random	0.45 (0.20, 1.01)	.054
TNM stage (I + II vs III + IV)	10	1141	82.70	< .0001	Random	0.31 (0.14, 0.68)	.003
Vascular invasion (Yes vs No)	4	398	85.90	< .0001	Random	4.97 (0.92, 26.89)	.063
Tumor differentiation (I + II vs III + IV)	9	1081	86.50	< .0001	Random	0.24 (0.08, 0.76)	.015
Cirrhosis (Yes vs No)	8	839	77.80	< .0001	Random	2.10 (1.00, 4.42)	.050
Tumor embolus (Yes vs No)	6	851	68.70	.007	Random	1.70 (0.76, 3.80)	.198

AFP = α-fetoprotein, CI = confidence interval, HBV = hepatitis B virus, OR = odds ratio, TNM = tumor node metastasis.

#### 3.2.1. The prognostic value of CD133 for OS

**Figure 2.**
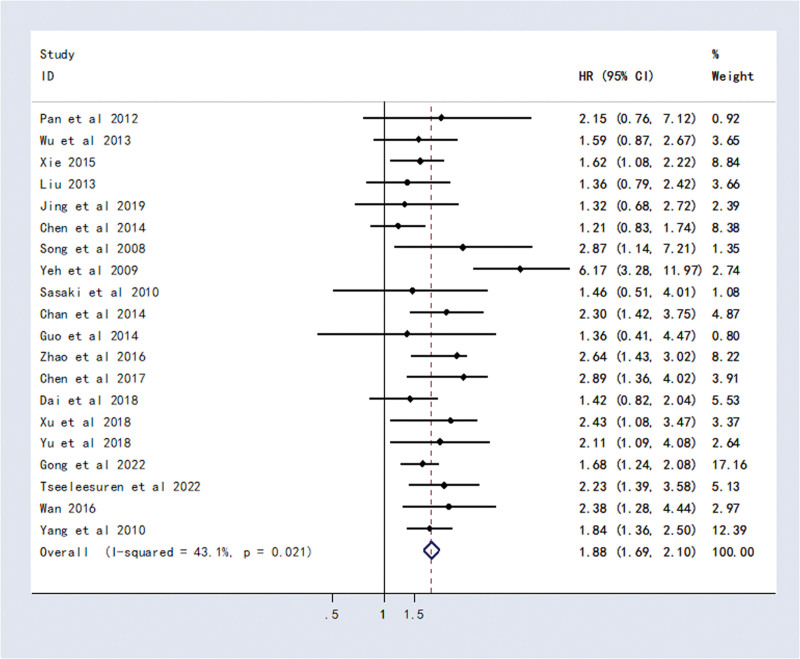
Forest plot of studies evaluating hazard ratios of high CD133 expression and the overall survival of HCC patients. CI = confidence interval, HCC = hepatocellular carcinoma, HR = hazard ratio.

Twenty studies found a link between CD133 and survival in HCC patients. Meta-analysis showed that patients with CD133-positive expression had a shorter OS than those with CD133-negative expression (HR = 1.88, 95% CI (1.69, 2.10), *P* < .0001) (Fig. 2).

#### 3.2.2. The prognostic value of CD133 for DFS

**Figure 3.**
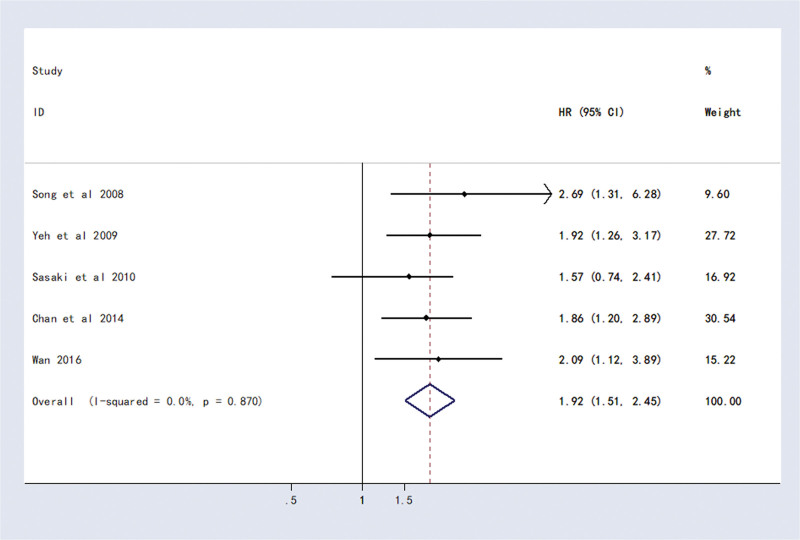
Forest plot of studies evaluating hazard ratios of high CD133 expression and the disease-free survival of HCC patients. CI = confidence interval, HCC = hepatocellular carcinoma, HR = hazard ratio.

Five studies reported the association of CD133 with DFS in HCC patients. Meta-analysis showed that patients with CD133-positive expression had shorter DFS than those with CD133-negative expression (HR = 1.92, 95% CI (1.51, 2.45), *P* < .0001) (Fig. 3).

#### 3.2.3. The prognostic value of CD133 for RFS

**Figure 4.**
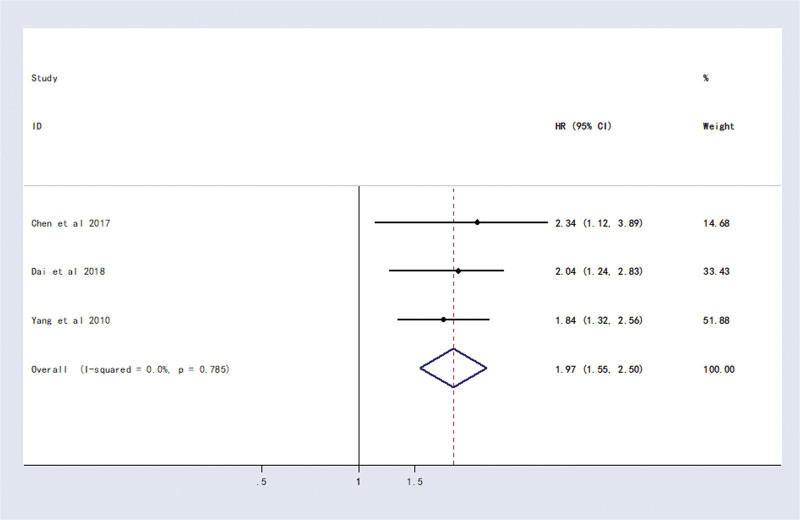
Forest plot of studies evaluating hazard ratios of high CD133 expression and the recurrence-free survival of HCC patients. CI = confidence interval, HCC = hepatocellular carcinoma, HR = hazard ratio.

Three studies reported the relationship between CD133 and RFS in HCC patients. Meta-analysis showed that patients with CD133-positive expression had a shorter RFS than those with CD133-negative expression (HR = 1.97, 95% CI (1.55, 2.50), *P < .0001*) (Fig. 4).

### 3.3. Subgroup Analysis

Subgroup analysis based on sample size (> 100 vs ≤ 100) statistical differences regardless of sample size > 100 or ≤ 100 (Table 3).

**Table 3:** Subgroup analysis of the relationship between CD133 and OS in patients with HCC.

Subgroup analysis	No. of studies	Heterogeneity		HR (95% CI)	*P*
		*I* ^*2*^	*P*		
Sample size					
>100	12	58.70%	.005	1.94 (1.67, 2.27)	< .0001
≤100	8	0.00%	.524	2.02 (1.65, 2.48)	< .0001
Treatment					
Surgery	18	37.30%	.056	1.95 (1.74, 2.19)	< .0001
Other	2	74.90%	.046	1.62 (0.89, 2.93)	0.117
Follow-up time					
≤ 60 months	2	42.60%	.187	1.89 (1.62, 2.20)	<.0001
> 60 months	9	68.30%	.001	1.83 (0.86, 3.88)	.115

CI = confidence interval, HCC = hepatocellular carcinoma, HR = hazard ratio, OS = overall survival.

Subgroup analyses carried out according to the type of treatment (surgery vs other) showed that positive expression of CD133 was associated with poor OS after HCC surgery (Table 3).

A subgroup analysis based on follow-up time (≤ 60 months vs > 60 months) showed that follow-up time was ≤ 60 months, and positive expression of CD133 was associated with poor OS in HCC (Table 3).

### 3.4. Sensitivity analysis

**Figure 5.**
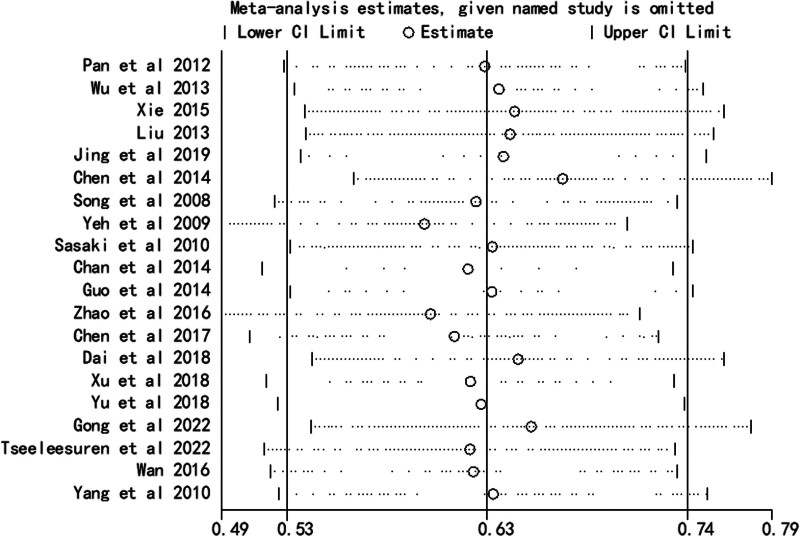
Result of sensitivity analysis for pooled overall survival estimation. CI = confidence interval.

Sensitivity analysis for OS showed that meta-analysis results were stable (Fig. 5).

### 3.5. Publication Bias

**Figure 6.**
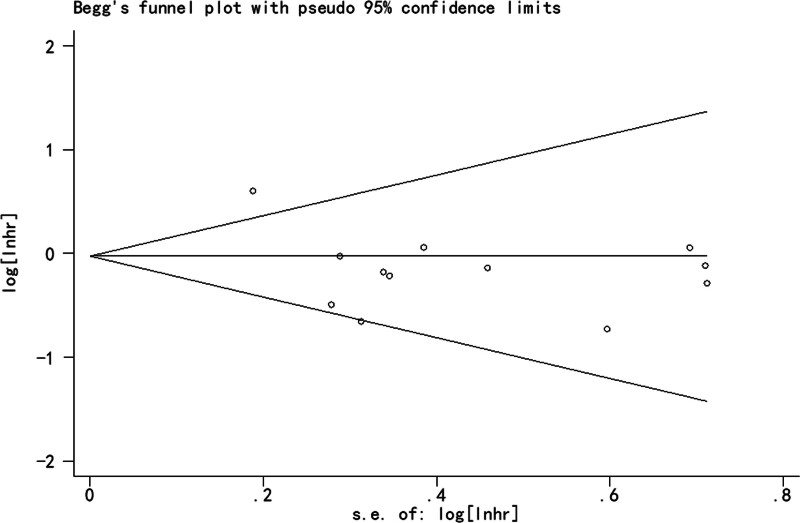
Funnel plot of publication bias for overall survival.

OS was analyzed for publication bias. The results showed no publication bias (Egger *P* = .933 and Begg *P *= .891) (Fig. 6).

## 4. Discussion

Due to the frequent recurrence and metastasis of HCC after treatment, it is a challenge for clinical treatment at present.^[62]^ Although multiple clinical studies^[28,29,31–36,45–52]^ have investigated the prognostic value of CD133 in HCC, published results have been inconclusive. CD133 is one of the characteristic markers on the surface of tumor stem cells. Its expression is of great value in tumor prediction, diagnosis, disease progression, metastasis, and prognosis. Therefore, it is necessary to clarify its prognostic value in HCC. Previous meta-analyseshave explored the prognostic role of CD133 in HCC.^[37,38]^ Compared with these earlier studies, this Meta-analysis offers important updates and advancements in the following aspects. Firstly, as far as we know, this study is the largest meta-analysis to date that specifically examines the prognostic value of CD133 in HCC. This significantly enhances the statistical power compared to previous analyses. Secondly, in addition to confirming the association between CD133 and OS and DFS, this analysis for the first time comprehensively and quantitatively demonstrated a significant association between CD133 positive expression and a worse RFS. Considering the high recurrence rate of HCC as an important endpoint indicator is crucial. Thirdly, we have updated the search time, making this study the most up-to-date evidence synthesis to date. Additionally, our subgroup analysis based on follow-up time has provided new insights into the role of CD133 as a predictor of 5-year survival. Therefore, this meta-analysis not only confirms the previous findings with stronger statistical power, but also provides new evidence for RFS and incorporates the latest literature, offering a more robust and timely evidence base for the prognostic value of CD133 in HCC.

CD133 is a member of the 5-time transmembrane glycoprotein prominin family that was originally used as a specific marker to screen human hematopoietic stem cells and progenitor cells.^[63]^ As a recognized marker of CSCs, it is used to specifically identify the source of different species of CSCs and diagnose diseases. CD133, as a functional molecule that can regulate tumor occurrence and development, plays a role in tumor recurrence, metastasis, and drug resistance. By activating multiple signaling pathways such as Wnt/-catenin and PI3K-AKT, the CD133 protein can affect tumor angiogenesis, hypoxia state, epithelial-mesenchymal transition, and other aspects. Eventually, it leads to the enhancement of tumor cell characterization and drug resistance.^[64–68]^

CD133 cells have been shown to be involved in HCC metastasis, tumorigenesis, tumor recurrence, and treatment resistance.^[69]^ The positive expression of CD133 was closely related to the clinicopathologic parameters of HCC, such as pathological grade and TNM stage.^[31,36,48]^ Our study found that CD133 was highly expressed in HCC tissues. Also, our study found that CD133 expression was associated with tumor size, TNM stage, and tumor differentiation. As a result, our meta-analysis discovered that CD133 was associated with poor HCC survival. Furthermore, positive expression of CD133 has been found to be an independent prognostic factor for survival and tumor recurrence in HCC patients.^[45,48]^ This suggests that CD133 is one of the potential tumor markers for HCC and can be used as a risk factor to evaluate the survival and prognosis of HCC. Immune escape is an important mechanism of recurrence and metastasis in tumor patients after treatment.^[70]^ High PD-L1 expression is significantly associated with a poor prognosis in HCC patients.^[71]^ There is increasing evidence that CSCs use immunosuppressive effects to evade recognition by the immune system.^[72–74]^ High levels of PD-L1 expression and CD44/CD133 are associated with poorer survival.^[72]^ Therefore, CD133 may be a marker worth investigating to predict the immunotherapeutic response in HCC. In addition, CD133 expression correlates with other markers of HCC stem cells. Pan et al^[35]^ showed that CD133 expression was positively correlated with EpCAM expression. A study by Xie^[51]^ showed that CD133 was significantly correlated with EpCAM expression, and as CD133 expression levels increased, EpCAM expression also increased. A study by Chen et al^[31]^ showed that dual positivity for CD44 and CD133 was an independent risk factor for the prognosis of HCC. A study by Zhao et al.^[61]^ also showed the same results. Therefore, it is important to combine different CSC markers to predict the prognosis of HCC patients.

In this study, subgroup analysis showed that sample size > 100, sample size ≤ 100, follow-up time ≤ 60 months, and surgery were related to shorter OS in HCC patients with CD133 positive expression. The primary treatment for HCC is surgery. Subgroup analysis found that positive CD133 expression was associated with a poor postoperative prognosis. However, other treatment methods, due to the limited literature, need further research to be explored. It is worth noting that Tseeleesuren et al^[49]^ showed that CD133 positive expression predicted survival after transcatheter arterial embolization/TACE treatment. In addition, overexpression of CD133 in HCC has been shown to be associated with poor response to sorafenib.^[75]^ HCC recurrence usually occurs 5 years after surgery. As a result, our subgroup analysis discovered that patients with a follow-up time of 60 months or less and CD133-positive HCC had a shorter OS. However, no such difference was observed in patients with a follow-up period exceeding 60 months. This may be related to the fact that most patients choose nonsurgical treatment after a recurrence of HCC. HCC has a high recurrence rate,^[7,8]^ with a 5-year survival rate of only 30–50%.^[9,10]^ Therefore, positive expression of CD133 may be an effective biomarker for predicting the 5-year survival of HCC patients.

There are some limitations to this meta-analysis. First of all, the positive criteria for CD133 are not uniform, which may lead to bias. Second, and most notably, as we have highlighted, all participants included in this meta-analysis were of Asian, with 23 out of 25 studies originating from China and 1 from Japan. This represents a significant limitation for the generalizability of our findings. Given the well-documented global variations in HCC etiology (e.g., hepatitis B virus vs hepatitis C virus dominance, alcohol related, or metabolic associated fatty liver disease), as well as potential differences in genetic backgrounds, environmental exposures, and clinicopathological characteristics, our conclusions (while robust for Asian populations) cannot be directly extrapolated to patient populations in other regions such as North America, Europe, or Africa. The prognostic value of CD133 in these diverse groups requires further independent investigation.. Third, this study only included literature published in Chinese and English, which may have a certain language bias. Fourthly, some HR included in the study did not directly give relevant outcome indicators, which need to be manually extracted from the survival curve and calculated, which may affect the accuracy of the results.

## 5. Conclusions

In summary, this meta-analysis suggested that high CD133 expression was associated with poor OS, DFS, and RFS in HCC patients. Overall, based on current findings, CD133 can be used as a predictor of prognosis in HCC patients. However, due to the predominant limitation of a single-ethnicity cohort and other factors mentioned above, large-scale, well-designed prospective studies involving diverse and multiethnic cohorts are urgently needed to validate these findings and establish the universal prognostic role of CD133 in HCC.

## Acknowledgments

The authors thank the authors of the included studies who shared the important data.

## Author contributions

**Conceptualization:** Dandan Liang.

**Data curation:** Baoguo Kang, Dandan Liang.

**Formal analysis:** Baoguo Kang, Dandan Liang.

**Funding acquisition:** Baoguo Kang, Dandan Liang.

**Investigation:** Baoguo Kang, Dandan Liang.

**Methodology:** Baoguo Kang, Dandan Liang.

**Project administration:** Dandan Liang, Dajie Liu.

**Resources:** Dajie Liu.

**Software:** Dajie Liu.

**Supervision:** Dajie Liu.

**Validation:** Baoguo Kang, Dandan Liang, Dajie Liu.

**Visualization:** Baoguo Kang, Dandan Liang, Dajie Liu.

**Writing – original draft:** Baoguo Kang, Dandan Liang, Dajie Liu.

**Writing – review & editing:** Baoguo Kang, Dandan Liang, Dajie Liu.

## Supplementary Material


