# Supplementary material for: Prognostic and clinicopathological significance of CD133 in patients with hepatocellular carcinoma: A meta-analysis
Source: Medicine (Baltimore). 2026 Apr 24;105(17):e48389. doi: 10.1097/MD.0000000000048389 (PMC13124420; doi:10.1097/MD.0000000000048389)
Supplement: Supplementary file 1 [file medi-105-e48389-s001.pdf]

Supplementary materials Table S1.Search strategy

#1	(CD133[Title/Abstract] OR prominin-1[Title/Abstract])
#2	("Hepatocellular Carcinoma"[Title/Abstract] OR "Liver Cancer"[Title/Abstract] OR HCC[Title/Abstract] OR liver neoplasms[Title/Abstract] OR"Hepatocellular Carcinoma"[MeSH terms])
#3	(prognos*[Title/Abstract] OR survival[Title/Abstract] OR clinicopath*[Title/Abstract]
#4	#1 AND #2 AND #3
